# Still's Disease and Recurrent Complex Regional Pain Syndrome Type-I: The First Description

**DOI:** 10.1155/2012/842564

**Published:** 2011-11-17

**Authors:** César Faillace, Jozélio Freire de Carvalho

**Affiliations:** Rheumatology Division, Clínica de Oncologia (CLION), Rua Altino Seberto de Barros, 119, 7 andar, 41810-570 Salvador, BA, Brazil

## Abstract

Complex regional pain syndrome (CRPS) is a chronic neuropathic pain disorder characterized by neuropathic pain associated with local edema and changes suggestive of autonomic involvement such as altered sweating, skin color, and skin temperature of the affected region. CRPS was described associated with several diseases, such as trauma, psychiatric conditions, and cancer. However, no case associated with Still's disease has been previously described. In this paper, the authors describe the first case of CRPS associated with Still's disease.

## 1. Introduction

Complex regional pain syndrome (CRPS), also known as reflex sympathetic dystrophy and causalgia, algodystrophy, Sudeck's atrophy, hand-shoulder syndrome, neuroalgodystrophy, and posttraumatic sympathetic dystrophy, is a chronic neuropathic pain disorder characterized by autonomic findings and typically develops in an extremity after acute tissue trauma. In addition to classic neuropathic pain characteristics (intense burning pain, hyperalgesia, and allodynia), CRPS is associated with local edema and changes suggestive of autonomic involvement (altered sweating, skin color, and skin temperature in the affected region). Trophic changes to the skin, hair, and nails and altered motor function (loss of strength, decreased active range of motion, and tremors) may also occur. CRPS is subdivided to CRPS-I (reflex sympathetic dystrophy) and CRPS-II (causalgia), reflecting the absence or presence of documented nerve injury, respectively [1]. 

Although CRPS was first described in isolation, it can be linked to several diseases, such as trauma [1], psychiatric conditions [2], and cancer [3]. However, no case associated with Still's disease has been previously described. 

Therefore, the objective of this study was to describe the first case of CRPS associated with Still's disease.

## 2. Case Report

A 50-year-old female began to complain in 2005 of polyarthritis of her knees, wrists, elbows, ankles, and hand metacarpophalangeal joints associated with fever, morning stiffness (for 4 hours), and evanescent rash. Laboratory results demonstrated leukocytosis, high levels of ferritin 401 ng/mL (reference value: 22–322 ng/mL), and erythrocyte sedimentation rate of 57 mm/1st hour. Antinuclear antibodies and rheumatoid factor were absent. Serologies for B and C hepatitis, HIV, HTLV 1 and 2, Epstein-Barr, rubella, toxoplasmosis, mononucleosis, rubella, and syphilis were negative. Echocardiography, liver and renal functions, myelogram, and bone marrow biopsy were also normal. A diagnosis of adult Still's disease was performed, and the patient was treated with nonsteroidal anti-inflammatory drugs and glucocorticoids. She evolved with no fever and improvement of polyarthritis; however, the knees, wrists, and elbows continued to be inflamed. Methotrexate (until 20 mg/week) was added to the scheme. She continued to have arthritis, sporadic fever, morning stiffness (for 2 hours), and leukocytosis (12,610). Treatment with infliximab (300 mg at 0, 2, and 6 weeks and then every 8 weeks, intravenously) was then initiated. She experienced no improvement after 6 months. Infliximab was then replaced by tocilizumab (8 mg/kg dose, monthly). She experienced marked improvement after this drug treatment. This approach also allowed reduction of the prednisone dose to 5 mg/day. In 2007, the patient received a diagnosis of carpal tunnel syndrome confirmed by electroneurography and was operated upon. In 2009, she noticed abrupt pain and edema in her right hand, clinical examination of which demonstrated cold swelling of the entire right hand and local diaphoresis. Thus, a diagnosis of complex regional pain syndrome type-I arthropathy was made. She was treated with prednisone 20 mg/day, NSAID, and physical therapy with improvement. She experienced five recurrences of CRPS, with good response to the therapeutic scheme outlined above. Currently, the patient is asymptomatic, with levels of ferritin at 21.5 ng/mL, CRP at <5 mg/L, and ESR at 3 mm/1st hour. The patient is also currently treated with tocilizumab monthly, prednisone at 2.5 mg/day, and methotrexate at 20 mg/week.

## 3. Discussion

This is the first description of the cooccurrence of CRPS in a patient with Still's disease.

Noxious events, including minor trauma, bone fracture, or surgery of the affected limb, often determine the onset of CRPS I. Occasionally, the disease develops after other medical events such as shoulder trauma, myocardial infarction, or a lesion of the central nervous system. In the present case, the patient had a previous carpal tunnel syndrome surgery performed at her wrist. In fact, several studies have demonstrated that the surgical stimulus may produce the clinical picture of CRPS. 

Regarding treatment, nonsteroidal anti-inflammatory drugs have not been demonstrated to have significant analgesic properties in CRPS. The use of opioids in CRPS has not been studied. Tricyclic antidepressants are the most well-studied medications in the context of neuropathic pain, and they have shown an analgesic effect. Glucocorticoids taken orally have clearly demonstrated efficacy in controlled trials [4]. There is no evidence that other immune-modulating therapies, notably intravenous immunoglobulins or immunosuppressive drugs, are effective in the treatment of CRPS. Subcutaneous calcitonin only had a mild effect on spontaneous pain [5]. However, bisphosphonates (alendronate, clodronate) induced significant improvement in pain, swelling, and movements [6].

Clinical experience and two prospective studies indicate that physiotherapy is of the utmost importance in achieving the recovery of function and rehabilitation [7, 8].

Inflammation may also play a role in this unique association of Still's disease and CRPS. In fact, an increased inflammatory response is an important pathophysiological mechanism in CRPS [9]. Indeed, some of the clinical features of CRPS, particularly in its early phase, could be explained by an inflammatory process [10]. Consistent with this idea, corticosteroids are often successfully used to treat acute CRPS [4]. There is increasing evidence that localized neurogenic inflammation might be involved in the generation of acute edema, vasodilatation, and increased sweating. Scintigraphic investigations using radiolabelled immunoglobulins show extensive plasma extravasation in patients with acute CRPS I [11]. Analysis of joint fluid and synovial biopsies in CRPS patients has revealed an increase in protein concentration, synovial hypervascularity, and neutrophil infiltration [12]. Furthermore, synovial effusion is enhanced in affected joints, as determined using MRI [13]. In acute untreated CRPS I patients, protein extravasation elicited by strong transcutaneous electrical stimulation was only provoked on the affected extremity compared with the normal side, indicating that substance P might be involved [14].

In summary, our case represents the first adult patient with Still's disease who had associated CRPS that recurred after hand surgery. Either this operation or the inflammation itself may have triggered CRPS development in this patient.
